# Polygenic risk score-based prediction of breast cancer risk in Taiwanese women with dense breast using a retrospective cohort study

**DOI:** 10.1038/s41598-024-55976-9

**Published:** 2024-03-15

**Authors:** Chih-Chiang Hung, Sin-Hua Moi, Hsin-I Huang, Tzu-Hung Hsiao, Chi-Cheng Huang

**Affiliations:** 1 Division of Breast Surgery, Department of Surgery, Taichung Veterans General Hospital, Taichung, 407 Taiwan; 2 Department of Applied Cosmetology, College of Human Science and Social Innovation, Hung Kuang University, Taichung, 433 Taiwan; 3 College of Life Sciences, National Chung Hsing University, Taichung, 402 Taiwan; 4 Graduate Institute of Clinical Medicine, College of Medicine, Kaohsiung Medical University, Kaohsiung, 807 Taiwan; 5 Research Center for Precision Environmental Medicine, Kaohsiung Medical University, Kaohsiung, 807 Taiwan; 6 Department of Medical Research, Kaohsiung Medical University Hospital, Kaohsiung Medical University, Kaohsiung, 807 Taiwan; 7 Department of Information Management, National Sun Yat-Sen University, Kaohsiung, 804 Taiwan; 8 International Integrated Systems, INC, Kaohsiung, 806 Taiwan; 9 Department of Medical Research, Taichung Veterans General Hospital, 1650 Taiwan Boulevard Sect. 4, Taichung, 407219 Taiwan; 10 Department of Public Health, Fu Jen Catholic University, New Taipei City, 242 Taiwan; 11 Institute of Genomics and Bioinformatics, National Chung Hsing University, Taichung, 402 Taiwan; 12 Division of Breast Surgery, Department of Surgery, Taipei Veterans General Hospital, Taipei, 112 Taiwan; 13 Institute of Epidemiology and Preventive Medicine, College of Public Health, National Taiwan University, No.17, Xuzhou Rd., Taipei City, 100 Taiwan

**Keywords:** Breast cancer, Dense breast, Polygenic risk score, Genotyping, Precision screening, Cancer genomics, Breast cancer, Cancer screening

## Abstract

Mammographic screening has contributed to a significant reduction in breast cancer mortality. Several studies have highlighted the correlation between breast density, as detected through mammography, and a higher likelihood of developing breast cancer. A polygenic risk score (PRS) is a numerical score that is calculated based on an individual's genetic information. This study aims to explore the potential roles of PRS as candidate markers for breast cancer development and investigate the genetic profiles associated with clinical characteristics in Asian females with dense breasts. This is a retrospective cohort study integrated breast cancer screening, population genotyping, and cancer registry database. The PRSs of the study cohort were estimated using genotyping data of 77 single nucleotide polymorphisms based on the PGS000001 Catalog. A subgroup analysis was conducted for females without breast symptoms. Breast cancer patients constituted a higher proportion of individuals in PRS Q4 (37.8% vs. 24.8% in controls). Among dense breast patients with no symptoms, the high PRS group (Q4) consistently showed a significantly elevated breast cancer risk compared to the low PRS group (Q1–Q3) in both univariate (OR = 2.25, 95% CI 1.43–3.50, *P* < 0.001) and multivariate analyses (OR: 2.23; 95% CI 1.41–3.48, *P* < 0.001). The study was extended to predict breast cancer risk using common low-penetrance risk variants in a PRS model, which could be integrated into personalized screening strategies for Taiwanese females with dense breasts without prominent symptoms.

## Introduction

Breast cancer is a major health concern in many countries, including Taiwan. According to the World Health Organization, 2.3 million women worldwide received a diagnosis of breast cancer in 2020^1^. The 2020 Taiwan Cancer Registry Annual Report indicated breast cancer as the most prevalent type of cancer among Taiwanese women, representing 28.5% of all newly diagnosed cases of cancer in this population (total number of new cases: 15,205). The incidence of breast cancer in Taiwan has exhibited a consistent upward trend over the past 3 decades (from 32.8 per 100,000 women in 1992 to 74.3 per 100,000 women in 2020), indicating its status as an increasing public health concern^2^. This trend underscores the importance of effective screening efforts. In July 2004, the Taiwanese government implemented a nationwide screening program involving biennial mammography for women aged 40–69. Such screening results in significant reductions in breast cancer-related mortality^3^. Asian females are more likely to have dense breast tissues than other women; mammography-detected breast density has been reported to be correlated with breast cancer risk^4^. Among Asian women, the risk of breast cancer increases with increasing breast density^5^.

From a clinical standpoint, multigene cancer predisposition panels and germline genetic testing serve as valuable tools that enables clinician to provide women with counseling regarding their individual risk of breast cancer. However, how to effectively translate genetic information into evidence-based clinical decisions remains unclear. Furthermore, the added benefits of breast cancer prevention and surveillance strategies personalized in accordance with carrier status for moderate penetrance genes are less clear than those of the strategies for high-penetrance genes, such as *BRCA1* and *BRCA2*^6^. A polygenic risk score (PRS) is a numerical score that is calculated on the basis of an individual's genetic data, specifically information on DNA sequence variants, to estimate the risk of a particular disease or condition. PRSs are typically derived from large-scale genetic studies, such as genome-wide association studies (GWAS), which identify genetic variants that are associated with a particular disease or trait^7^. To calculate a PRS, genetic variants that have been associated with the disease or trait of interest are combined and weighted according to their effect sizes, which represent the strength of the association between the genetic variant and the disease or trait. Then, the weighted genetic variants are summed to generate an overall score that represents an individual's genetic risk for the disease or trait^8,9^. PRS can be used for various purposes, such as the prediction of an individual's risk of developing a disease, risk-based stratification of individuals, and identification of high-risk individuals who may benefit from early screening or targeted prevention strategies^10^.

PRS may reveal low-penetrance risk variants profiles in individuals for specific disease. However, the use of a PRS for estimating breast cancer risk is typically restricted to gene-only models, and PRSs are rarely integrated with breast cancer screening (BCS) databases. BCS databases encompass a wide range of risk factors closely linked to breast cancer development. In this study, we evaluated the ability of a PRS to predict breast cancer risk in women with dense breasts. In addition, we investigated whether certain single nucleotide polymorphisms (SNPs) can serve as the indicators of breast cancer risk. Furthermore, we calculated the PRSs of our study cohort to identify genetic factors associated with clinical characteristics in females at-risk and the development risk of breast cancer.

## Results

The clinical characteristics of the study cohort are summarized in Table 1. The mean age of the study cohort was 57.4 ± 7.6 (range: 40–71) years. Most of the included women were well-educated, with > 70% having received at least high school education. The cohort included 6315 women. Of them, 8.9% had a family history of breast cancer: 3.3% had a first-degree relative with breast cancer, and 5.6% had a second-degree relative with the disease. Approximately, 87% of the women had a history of pregnancy, with the average age at first birth being 26.6 ± 4.5 years. Breast cancer (27.6 ± 4.7 years) had a later age at first birth compared to non-breast cancer controls (26.5 ± 4.5 years, *P* = 0.023). The pregnancy parity in the study cohort was mostly one to two, and approximately 46.1% had experience with breastfeeding. Of the study cohort, 75.7% reported experiencing menopause, while breast cancer (65.8%) exhibited a significantly lower proportion in menopausal compared to controls (75.9%, *P* = 0.014). However, the age at menopause between both subgroups showed no significant difference, mostly around 49.6 ± 5.0 years. Approximately 10.3% and 13.0% of the study cohort reported previous use of oral contraceptive and hormone replacement therapy, respectively. In addition, 7.4% study cohort had a history of breast surgery. Furthermore, approximately 33% women had benign breast disease, including non-proliferative or proliferative without atypia. Only 18.6% women reported regularly conducting self-examinations. Approximately 6.6% of the included women had mastalgia or palpable breast lesions and 7.3% had a history of any other type of cancer. Regarding diagnostic evaluations, 28.5% of the women had undergone mammogram once and 12.6%, twice. The prevalence of breast cancer family history, breast surgery history, and breast symptoms was higher among women with breast cancer than among control (non-breast cancer) individuals. Notably, the prevalence of mastalgia or palpable breast lesions was significantly higher among women with breast cancer than among the control individuals (*P* < 0.001).

**Table 1:** Clinical characteristics of study cohort (n = 6335).

Characteristics	Overall	Controls	Breast cancer	*P*
No. of patients	6335	6224	111	
Age at mammography	57.4 ± 7.6	57.4 ± 7.6	56.7 ± 7.3	0.337
Education				0.732
Without education	67 (1.1)	66 (1.1)	1 (0.9)	
Primary	752 (11.9)	743 (11.9)	9 (8.1)	
Secondary	803 (12.7)	787 (12.6)	16 (14.4)	
High/professional	2024 (31.9)	1990 (32.0)	34 (30.6)	
Bachelor	2683 (42.4)	2632 (42.3)	51 (45.9)	
Refused to answer	6 (0.1)	6 (0.1)	–	
*Breast cancer family history*				
Overall	565 (8.9)	552 (8.9)	13 (11.7)	0.298
First-degree relative	208 (3.3)	205 (3.3)	3 (2.7)	1.000
Second-degree relative	354 (5.6)	344 (5.5)	10 (9.0)	0.113
Age at menarche	13.8 ± 1.5	13.8 ± 1.5	13.7 ± 1.3	0.157
*Reproductive factors*				
Previous pregnancy	5509 (87.0)	5412 (87.0)	97 (87.4)	0.893
Age at first birth	26.6 ± 4.5	26.5 ± 4.5	27.6 ± 4.7	**0.023**
Parity				–
1	766 (12.1)	746 (12.0)	20 (18.0)	
2	2589 (40.9)	2551 (41.0)	38 (34.2)	
≥ 3	2154 (34.0)	2115 (34.0)	39 (35.1)	
Breast feeding	2922 (46.1)	2875 (46.2)	47 (42.3)	0.420
Menopause	4796 (75.7)	4723 (75.9)	73 (65.8)	**0.014**
Age at menopause	49.6 ± 5.0	49.6 ± 5.0	49.8 ± 5.5	0.737
Oral contraceptive (OC)	653 (10.3)	636 (10.2)	17 (15.3)	0.080
Hormone replacement therapy (HRT)	824 (13.0)	809 (13.0)	15 (13.5)	0.873
Breast surgery history	470 (7.4)	458 (7.4)	12 (10.8)	0.169
Benign breast disease	2088 (33.0)	2058 (33.1)	30 (27.0)	0.180
Self-exam experience	1181 (18.6)	1160 (18.6)	21 (18.9)	0.940
Breast syndrome				**< 0.001**
No symptoms	5914 (93.4)	5834 (93.7)	80 (72.1)	
Mastalgia/palpable	421 (6.6)	390 (6.3)	31 (27.9)	
Other cancer history	463 (7.3)	459 (7.4)	4 (3.6)	0.130
Mammogram				–
None	3729 (58.9)	3634 (58.4)	95 (85.6)	
1 episode	1808 (28.5)	1794 (28.8)	14 (12.6)	
2 episodes	798 (12.6)	796 (12.8)	2 (1.8)	

Significant p-values are in bold.

**Figure 1:**
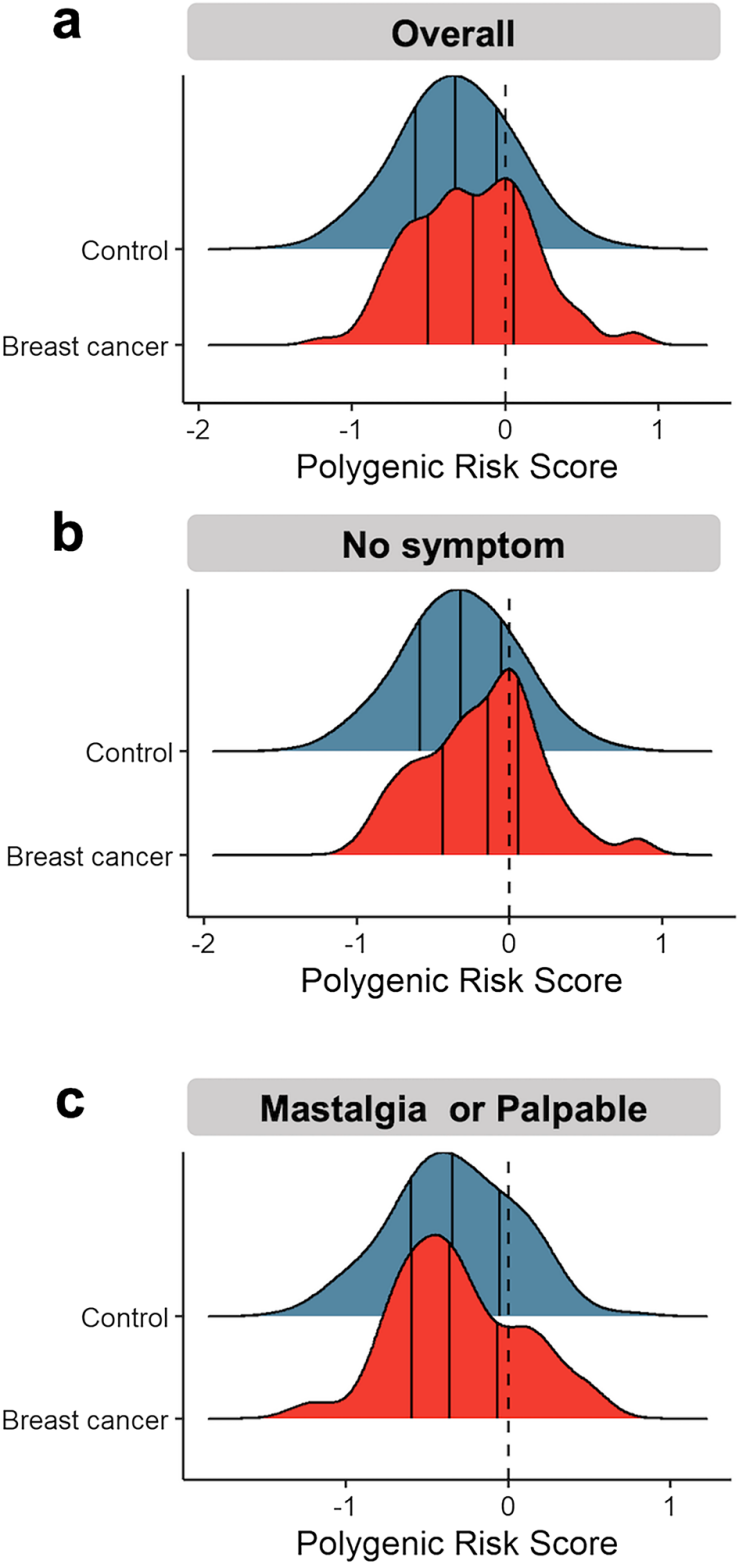
Distribution of PRS in the study cohort. (**a**) In the overall cohort, (**b**) among women with no breast symptoms, and (**c**) among women with mastalgia or palpable lesion.

The distribution of PRSs in the study cohort is depicted in Fig. 1a. Patients with breast cancer had higher PRS than did control individuals. We further divided the cohort into four quartiles (Q1–Q4; Table 2) and found that the proportion of women with breast cancer in PRS Q4 was higher than that of control individuals (37.8% vs. 24.8%, respectively). The results of the analysis indicated that the risk of breast cancer in PRS Q4 was significantly higher than that in PRS Q1 (OR: 1.93; 95% CI 1.16–3.31; *P* = 0.013); however, no significant difference in breast cancer risk was noted between PRS Q1 and PRS Q2 or Q3. We further divided the patients into a high-PRS (PRS Q4) and a low-PRS (PRS Q1–Q3) subgroups; the risk of breast cancer was significantly in the high-PRS subgroup than in the low-PRS subgroup (OR: 1.85; 95% CI 1.25–2.71; *P* = 0.002). Additionally, we conducted multivariate analyses for both PRS subgroups, adjusting for breast cancer-associated risk factors, including previous pregnancy, menopause status, oral contraceptive use, and hormone replacement therapy. These results remained consistent with the univariate analysis, demonstrating that after adjusting for these relevant risk factors, the PRS continued to exhibit robust contributions to breast cancer risk estimation.

**Table 2:** Association of the PRS quartiles in breast cancer risk.

Characteristics	Overall	Controls	Breast cancer	OR (95% CI)^a^	*P*	OR (95% CI) ^b^	*P*
PRS quartile							
Q1	1584 (25.0)	1562 (25.1)	22 (19.8)	Referent		Referent	
Q2	1584 (25.0)	1559 (25.0)	25 (22.5)	1.14 (0.64, 2.04)	0.700	1.13 (0.64, 2.04)	0.668
Q3	1583 (25.0)	1561 (25.1)	22 (19.8)	1.00 (0.55, 1.82)	1.000	0.99 (0.54, 1.80)	0.973
Q4	1584 (25.0)	1542 (24.8)	42 (37.8)	1.93 (1.16, 3.31)	**0.013**	1.93 (1.16, 3.30)	**0.013**
PRS subgroup							
Low (Q1–Q3)	4751 (75.0)	4682 (75.2)	69 (62.2)	Referent		Referent	
High (Q4)	1584 (25.0)	1542 (24.8)	42 (37.8)	1.85 (1.25, 2.71)	**0.002**	1.85 (1.25, 2.72)	**0.002**

Significant p-values are in bold.

*OR* odds ratio, *CI* confidence interval.

^a^Unadjusted results were estimated using univariate analysis.

^b^Results were adjusted for relevant factors, including previous pregnancy, menopause status, oral contraceptive use, and hormone replacement therapy.

The predictive performance of the PRS and breast cancer–related clinical characteristics were assessed (Table 3). The risk of breast cancer was significantly higher in the high-PRS subgroup than in the low-PRS subgroup; this result was obtained using both univariate and multivariate models. Notably, the model including breast cancer risk–associated clinical characteristics outperformed the PRS-only model; the OR increased from 0.565 (95% CI 0.520–0.611) to 0.727 (95% CI 0.677–0.777) after incorporation of clinical characteristics and relevant factors in our statistical model. The risk of breast cancer was significantly low in patients with benign breast disease (OR: 0.51; 95% CI 0.31–0.81; *P* = 0.006) and menopause (OR: 0.48; 95% CI 0.26–0.88; *P* = 0.018), but significantly high in those with mastalgia or palpable breast lesion (OR: 7.03; 95% CI 4.38–11.1; *P* < 0.001).

**Table 3:** Predictive performance of PRS for breast cancer risk.

Characteristics	Comparison	Univariate		Multivariate	
		OR (95% CI)	*P*	OR (95% CI)	*P*
Overall (n = 6335)					
PRS subgroup	High versus low	1.85 (1.25, 2.71)	**0.002**	1.86 (1.25, 2.75)	**0.002**
Age at mammography	Years	0.99 (0.96, 1.01)	0.356	1.03 (0.99, 1.07)	0.116
Family history of BC	Yes versus no	1.36 (0.72, 2.36)	0.299	1.44 (0.76, 2.52)	0.226
Previous pregnancy	Yes versus no	1.04 (0.61, 1.91)	0.893	1.14 (0.65, 2.13)	0.671
Breast surgery history	Yes versus no	1.53 (0.79, 2.69)	0.172	1.68 (0.82, 3.21)	0.134
Benign breast disease	Yes versus no	0.75 (0.48, 1.13)	0.181	0.51 (0.31, 0.81)	**0.006**
Self-exam experience	Yes versus no	1.02 (0.61, 1.61)	0.940	1.43 (0.85, 2.33)	0.163
Breast symptoms	Yes versus no	5.80 (3.73, 8.79)	** < 0.001**	7.03 (4.38, 11.1)	** < 0.001**
Others cancer	Yes versus no	0.47 (0.14, 1.12)	0.139	0.42 (0.13, 1.04)	0.099
Age at menarche	Years	0.90 (0.78, 1.04)	0.194	0.90 (0.77, 1.04)	0.173
Menopause	Yes versus no	0.61 (0.41, 0.92)	**0.015**	0.48 (0.26, 0.88)	**0.018**
OC	Yes versus no	1.59 (0.91, 2.61)	0.083	1.59 (0.91, 2.65)	0.087
HRT	Yes versus no	1.05 (0.58, 1.75)	0.873	1.02 (0.56, 1.73)	0.957
Harrel’s C-index (95% CI)		0.565 (0.520–0.611)^a^		0.727 (0.677–0.777)^b^	
No symptom (n = 5914)					
PRS subgroup	High versus low	2.25 (1.43, 3.50)	**< 0.001**	2.23 (1.41, 3.48)	**< 0.001**
Age at mammography	Years	0.99 (0.96, 1.02)	0.531	1.04 (1.0, 1.09)	0.087
Family history of BC	Yes versus no	1.31 (0.61, 2.49)	0.454	1.30 (0.60, 2.49)	0.470
Previous pregnancy	Yes versus no	1.15 (0.61, 2.49)	0.689	1.20 (0.61, 2.63)	0.622
Breast surgery history	Yes versus no	1.31 (0.55, 2.68)	0.495	1.81 (0.71, 4.03)	0.170
Benign breast disease	Yes versus no	0.67 (0.39, 1.10)	0.128	0.57 (0.31, 0.99)	0.054
Self-exam experience	Yes versus no	1.43 (0.85, 2.33)	0.159	1.43 (0.85, 2.34)	0.163
Others cancer	Yes versus no	0.48 (0.12, 1.30)	0.219	0.47 (0.11, 1.30)	0.210
Age at menarche	Years	0.93 (0.78, 1.07)	0.416	0.94 (0.78, 1.08)	0.468
Menopause	Yes versus no	0.55 (0.35, 0.88)	**0.011**	0.37 (0.18, 0.76)	**0.007**
OC	Yes versus no	1.70 (0.89, 2.99)	0.084	1.72 (0.90, 3.04)	0.080
HRT	Yes versus no	0.76 (0.33, 1.48)	0.457	0.75 (0.33, 1.48)	0.446
Harrel’s C-index (95% CI)		0.589 (0.534–0.644)^a^		0.682 (0.623–0.741)^b^	

Significant p-values are in bold.

*BC* breast cancer, *OC* oral contraceptive use, *HRT* hormone replacement therapy.

^a^Harrel’s C-index for PRS subgroup in univariate model.

^b^Harrel’s C-index for multivariate model with all retained variables.

Because women with breast cancers differed significantly from control individuals in term of clinical presentations, we further analyzed the distribution of PRSs in individuals stratified by breast symptoms. Overall, in women with no apparent breast symptoms, the distribution of exhibited a right-sided and narrow peak; their PRSs were higher than those of control individuals (Fig. 1b). However, when analyzing women with mastalgia or palpable lesion (421 patients), we did not observe the same right-sided and narrow pattern of PRS distribution (Fig. 1c). The results presented in Fig. 1b, c suggest that the association between PRS and breast cancer risk varies depending on the presence of specific breast clinical characteristics. Individuals without breast symptoms may be overlooked, or their risk of breast cancer may be underestimated; consequently, they may be less likely to be screened for breast cancer. Therefore, we conducted a subgroup analysis of women without breast symptoms to assess the performance of PRS model in predicting the risk of breast cancer in women with dense breasts but no symptoms. For this population, the predictive performance of the PRS-only model was 0.589 (95% CI 0.534–0.644) and that of the multivariate model (PRS plus clinical characteristics and relevant factors) was 0.682 (95% CI 0.623–0.741). Among 5914 women with dense breast but no symptoms, those with high PRSs (Q4) consistently had significantly higher risks of breast cancer than did women with low PRS groups (Q1–Q3); this finding was obtained using both univariate (OR: 2.25; 95% CI 1.43–3.50; *P* < 0.001) and multivariate analyses (OR: 2.23; 95% CI 1.41–3.48; *P* < 0.001). Furthermore, patients with menopausal status (OR: 0.37; 95% CI 0.18–0.76; *P* = 0.007) consistently showed a decreased breast cancer risk.

## Discussion

Our study findings revealed that the association between PRS and breast cancer risk may vary depending on the presence of specific breast symptoms and clinical characteristics. Because women without breast symptoms and clinical characteristics may be neglected in screening, a PRS may improve risk prediction when screening these individuals.

The incorporation of cancer-related genetic variants in a PRS model may help evaluate individuals’ genetic susceptibility to cancer^11,12^. In a study investigating the association between individuals’ PRSs and breast cancer risk, cancer susceptibility was determined on the basis of SNPs in women carrying pathogenic mutations in *BRCA1* and *BRCA2*^13^. The aforementioned study indicated that the incorporation of PRSs into risk prediction models can improve the calculation of personalized risk estimates for individuals carrying mutations in *BRCA1* and *BRCA2*; in addition, it can guide clinical decisions regarding the management of cancer risk^13^. Incorporating PRS calculation into breast cancer risk estimation may help health-care providers identify high-risk individuals and implement effective prevention and early detection strategies, such as early and frequent screenings and preventative interventions^11^.

A key point addressed in the present study is the potential effects of integrating PRS evaluation into screening protocols for estimating the risk of breast cancer. In a study that compared the use of family history data and PRSs for biennial screening in individuals aged 50–74 years, the combined method resulted in the highest gain in life-years (29%) and averted breast cancer related deaths^14^. These findings suggest that combining the evaluation of PRSs with the assessment of other risk factors, such as family history of breast cancer, is beneficial. We found that PRS evaluation has the potential to improve the prediction of breast cancer risk in women with dense breasts but no prominent symptoms; therefore, combining the evaluation of PRSs with the assessment of breast disease history and breast symptoms may also contribute to early detection of breast cancer.

More Asian women than Western women have dense breasts^15^. In this study, < 1% of all included women were diagnosed as having fatty breasts in our initial cohort. Dense breast tissue is a normal and common finding on mammograms and refers to the presence of breasts with higher proportions of glandular and connective tissues than that of fatty tissue^16^. The appearance of dense breast tissue is determined through mammography because no physical signs or symptoms are associated with such tissue^17^. No direct relationship has been reported between dense breasts and mastalgia or palpable lesions^18^. Although our findings indicate that women with dense breasts but no symptoms have PRSs hat differentiate them from those with mastalgia or palpable lesions, insufficient evidence precludes a determination of whether genetic differences exist between women with mastalgia or palpable lesions and those without these conditions. Nonetheless, women with dense breasts are at higher risk for breast cancer; supplementary screening tests may be necessary for early cancer detection and treatment in this population^17,19^. Therefore, personalized screening strategies may facilitate the prevention and early detection of breast cancer, particularly in high-risk women with dense breasts^19^.

The use of multiple-ancestry PRSs leverages genetic ancestral composition to extend the applicability of polygenic risk prediction beyond European populations, offering women of diverse and mixed ancestries with an opportunity to receive additional personalized treatment^20^. Hence, incorporating PRS evaluation into clinical screening involves the consideration of potential ethical and social issues pertaining to genetic testing and patient privacy. In addition, PRS evaluation may not be equally effective for all women, particularly those from non-European populations, among whom the genetic architecture of breast cancer may differ.

A limitation of this study was that it was conducted using data from a single institute, which limits the generalizability of our findings. Further research is needed to evaluate the optimal use of PRSs in clinical practice—the potential benefits and risks associated with incorporating PRS into breast cancer screening programs. In the future, large-scale, multicenter studies should be conducted incorporating PRS evaluation into breast cancer screening and prevention strategies; such research may reveal the predictive value of PRS in different populations. In addition, studies should be conducted to identify the best approaches for communicating PRS results to patients and healthcare providers. Despite the aforementioned limitations, our study highlights the potential benefits of incorporating PRS evaluation into programs for breast cancer risk prediction and management, particularly in populations that may be underrepresented or overlooked in traditional risk assessment methods. Further research is needed to explore the optimal use of PRSs in clinical practice to fully understand its role in breast cancer prevention and treatment. This study emphasized the potential benefits of using PRSs in the prediction of breast cancer risk in women with dense breasts, a group that may not manifest prominent symptoms. Integrating the evaluation of PRS with the assessment of menopause and breast symptoms may significantly contribute to the early detection of breast cancer.

## Conclusion

Breast cancer screening has undergone a shift from a general approach to a personalized, risk-based approach. Identifying the key factors that contribute to breast cancer susceptibility can help us develop screening protocols based on age, breast density, and other factors. Unlike the contemporary breast cancer screening guidelines that recommend the use of family history as the only risk factor, our proposed model identifies both genetic and clinical risk factors using big data analysis, with inputs from electronic health records, cancer screening data, and the cancer registry database of a single center. We extended breast cancer risk prediction by using common low-penetrance risk variants and constructing a PRS model, which could be integrated into personalized screening strategies for Taiwanese women with dense breasts without prominent symptoms. However, the clinical utility of PRSs in guiding breast cancer screening and prevention remains to be comprehensively established; therefore, further research is needed to determine the optimal use of PRSs in clinical practice.

## Methods

### Study cohort

**Figure 2:**
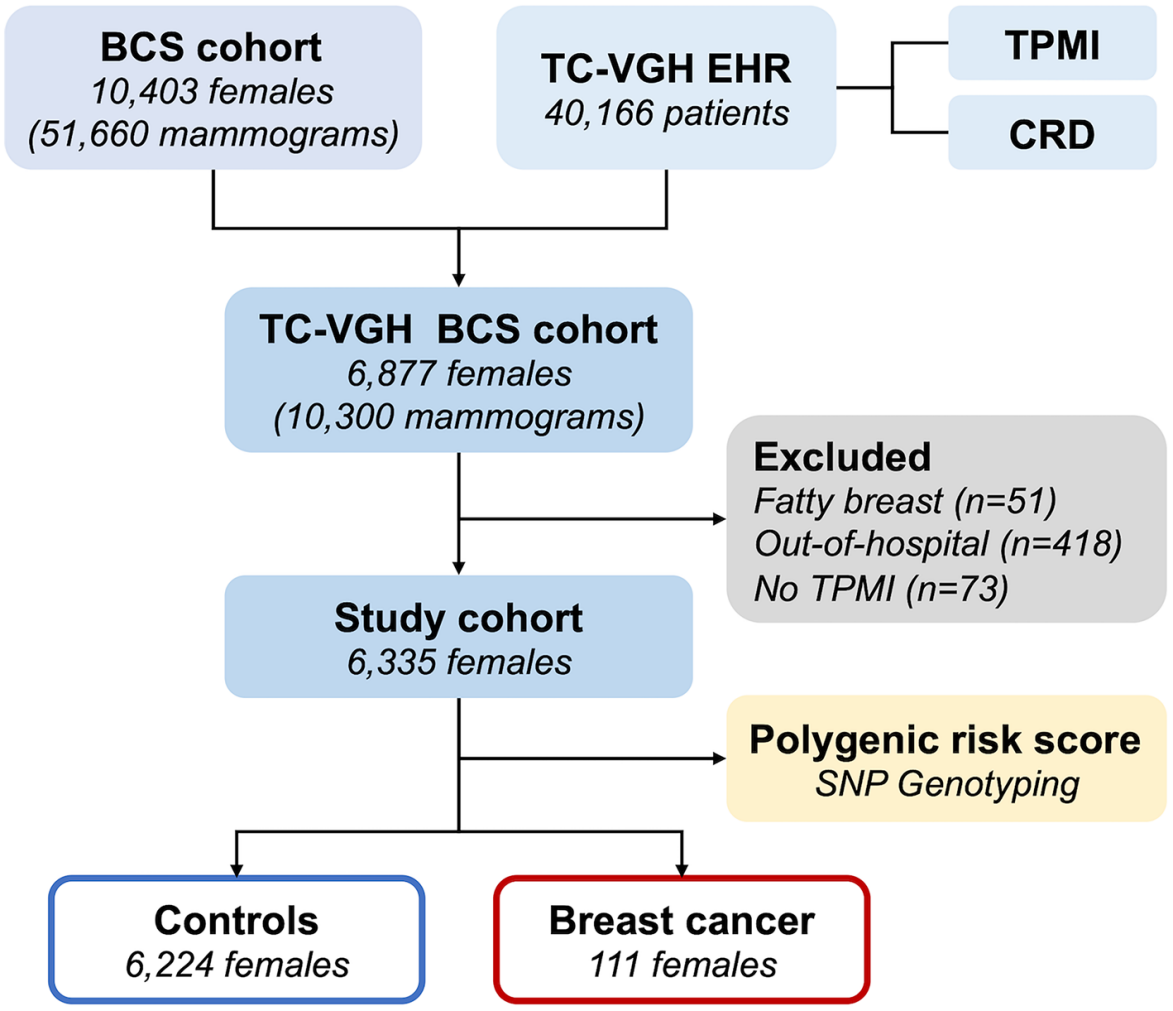
Study flowchart. *BCS* breast cancer screening, *TC-VGH EHR* Taichung Veterans General Hospital electronic health records, *TPMI* Taiwan Precision Medicine Initiative, *CRD* cancer registry database.

This retrospective cohort study was approved by the ethics committee of our institution (registered number CE23245B). All methods were performed in accordance with the relevant guidelines and regulations. The inclusion criteria were being women, having undergone mammography screening, and not meeting any of the following exclusion criteria: having fatty breasts, being screened in an out-of-hospital setting, and lacking Taiwan Precision Medicine Initiative (TPMI) genotyping data. The study flowchart is presented in Fig. 2. From the local BCS database, we retrieved the data of 10,403 females who undergone mammography between February 2017 and November 2022. Subsequently, we linked the BCS cohort to the electronic health records of Taichung Veterans General Hospital (TC-VGH) to obtain TPMI genotyping (registered number: SF19153A) and cancer registry data. In total, 10,403 local women who had undergone mammography were linked to the TC-VGH electronic health records database, which contained the data of 40,166 women. Of the 10,403 women, 6877 women were successfully mapped to the BCS cohort. We excluded 51 women with fatty breast, 418 women screened in an out-of-hospital setting (resulting in a lack of study information), and 73 women without TPMI genotyping data. Finally, a total of 6335 women were included in this study. Of them, 111 women received a diagnosis of breast cancer during the study period; the remaining women who were at risk of breast cancer were included as control individuals.

### Polygenic risk score (PRS)

The PRSs of the study cohort were estimated using TPMI genotyping data. To evaluate the PRSs, 77 SNPs were selected as candidates, all previously identified as breast cancer susceptibility loci for various types of breast cancer, and all SNPs have reached genome-wide significance (*P* < 5 × 10^−8^)^21^. The polygenic risk effects of these 77 candidate SNPs on breast cancer have been previously validated through a large European cohort study, leading to the establishment of a reported trait for breast cancer named PGS000001 (PGS Name: PRS77_BC) The candidate SNPs used in this study are listed in Supplementary Table S1. The PRS for 77 SNPs was derive using the Eq. (1).

1 $${\text{PRS}} = { }\mathop \sum \limits_{n = 1}^{77} {\upbeta }_{n} x_{n}$$

where ${\upbeta }_{n}$ is the per-allele log odds ratio (OR) estimated using logistic regression for breast cancer risk associated with SNP_*n*_, and ${x}_{n}$ is the allele dosage for SNP_*n*_. The distribution of PRS in the study cohort is illustrated using a ridge plot. The PRSs of the included individuals were divided into four quartiles, and the risk of breast cancer in each PRS quartile was estimated through binomial logistic regression.

### Statistical analysis

The clinical characteristics of the study cohort are presented using mean ± standard deviation or frequency and percentage values. The distribution of characteristics between women with breast cancer and control individuals were assessed using independent two-sample *t*-test, chi-square, and Fisher’s exact test. The associations of the PRS and relevant characteristics with the risk of breast cancer in the study cohort were investigated through univariate and multivariate binomial logistic regression. Harrel’s C-index was used to evaluate the predictive performance of PRS in both univariate and multivariate models. All *P* values were two-sided, and a *P* value < 0.05 was considered to be statistically significant. All analyses were performed using R 4.1.2 (R core team, 2023).

### Ethics approval and consent to participate

This study was approved by the Institutional Review Board of Taichung Veterans General Hospital (TC-VGH), Taichung, Taiwan (CE23245B and SF19153A). The breast cancer screening and cancer registry database were retrospectively obtained from electronic health records of TC-VGH under CE23245B approved protocol with the waiver of informed consent. The TPMI genotyping for polygenic risk score estimation was obtained from the TPMI database under SF19153A approved protocol with informed consents.

### Supplementary Information

Supplementary Information.

## Data Availability

The data supporting the findings of this study are available from the corresponding author upon reasonable request. We define reasonable as a request to review data for purposes of further study or clarification of any data or analyses presented in the paper.

## Abbreviations

PRS: Polygenic risk score
GWAS: Genome-wide association studies
BCS: Breast cancer screening
SNP: Single nucleotide polymorphisms
TPMI: Taiwan Precision Medicine Initiative
